# Adaptive or maladaptive music-listening coping strategy: How does neuroticism use music after experiencing a romantic relationship breakup?

**DOI:** 10.1371/journal.pone.0331373

**Published:** 2025-08-29

**Authors:** Hanwei He, Shu Chen, Cheng Hua

**Affiliations:** 1 Faculty of Music, Fujian Normal University, Fuzhou, Fujian, China; 2 Faculty of Music and Dance, China West Normal University, Nanchong, Sichuan, China; 3 Graduate School of Teaching, Leiden University, Leiden, Netherlands; University of Macerata: Universita degli Studi di Macerata, ITALY

## Abstract

Romantic relationship breakups are common experiences among young people and are associated with negative emotions that can jeopardize psychological well-being. Additionally, those with higher neuroticism may have greater emotional sensitivity towards the impact of music, as they tend to exhibit more negative and unstable emotional traits. As a result, they may be more inclined to control emotions by using music as a coping strategy. However, existing literature has rarely explored the differential roles of adaptive and maladaptive music-listening coping, as well as the mechanisms linking neuroticism and negative emotions associated with romantic breakups. To fill these gaps, the current study recruited 389 college students who had experienced at least one romantic breakup lasting two months or longer within the past five years. Participants completed a battery of questionnaires assessing relevant variables. Correlational analyses indicated that neuroticism and negative emotions related to romantic breakups were positively correlated with maladaptive music-listening coping. Conversely, neuroticism was negatively correlated with adaptive music-listening coping, while no discernible connection between adaptive music listening coping and negative emotions evoked by romantic breakups. Structural equation modeling suggested that neuroticism predicted emotional response to breakups through maladaptive music-listening coping, rather than adaptive music-listening coping. Theoretical and practical implications were discussed.

## Introduction

Relationship breakups are considered to be common experiences among young people in their 20s [1,2], as emerging adult relationships are often ephemeral [3]. Meanwhile, breakups are regarded among the most excruciating events in life [4]. Multiple research indicate that breakups correlate with many adverse effects, including anxiety irritability [5], heightened levels of depression [6], and even drug-alcohol abuse and suicidal ideation [7,8]. The high prevalence of breakups among young people and their negative effects render it necessary to investigate their antecedents and consequences.

Many researchers have suggested that personality factors are one of the main causes of breakups, with neuroticism being the most frequently mentioned personality [9–12]. Neuroticism is one of the dimensions of the Big Five personality model, used to describe individuals who vary in emotional stability. Those high in neuroticism are more likely to suffer from emotional instability, heightened sensitivity, and more intense and prolonged emotional reactions [10,13]. Meanwhile, negative emotions including anxiety, despair, vulnerability, and aggression were linked to neuroticism [14,15]. In other words, those who exhibit greater degrees of neuroticism are more prone to complain towards their partnership and perceive their lover’s behaviors negatively [16]. Therefore, neurotic individuals may show more distress following romantic breakups [17].

While the relationship between neuroticism and negative emotions following romantic breakups is well-documented, the underlying mechanism remains unclear. Given that individuals often rely on coping strategies to regulate their emotions, it is important to explore whether these strategies mediate the link between neuroticism and emotional distress after a breakup. Coping refers to strategies for consciously managing stress and involves proactive efforts to adjust emotions, cognitions, behaviors, and physiological responses in response to stressors [18]. According to stress and coping theory, people tend to have a variety of individualized coping strategies to regulate the negative effects of stress [19]. Studies have already found that during periods of high stress or negative emotions, individuals with neurotic traits often choose to listen to music as a means to adjust challenging emotional states [20,21]. However, coping does not always lead to positive outcomes [22]. In the case of music listening, it can be beneficial when it serves as a distraction, redirecting attention toward unrelated activities. Conversely, it may have negative effects when it enables neurotic individuals to escape or ruminate excessively, trapping them in their thoughts without resolution [23].

However, it is not yet known whether music listening, a coping strategy frequently used by neurotic individuals, is adaptive or maladaptive when they experience negative emotions. It is, therefore, necessary to identify which type of coping strategies exacerbate or mitigate their negative reactions following romantic breakups. Gaining this understanding could help promote adaptive coping mechanisms while minimizing maladaptive responses, especially among neurotic individuals.

### Neuroticism and negative emotions

Several studies have examined the connection between neuroticism and negative emotions, with a consistent positive relationship usually found between the two. For example, a study of emotional experiences over a seven-day period asked participants with neuroticism traits to report the positive and negative emotional experiences they had experienced that day by writing a diary at the end of each day’s activities. The results showed that neuroticism was positively correlated with the duration and frequency of negative emotions, and that the frequency of negative emotions emerged as the strongest predictor of neuroticism trait strength [24]. A longitudinal study by Caspi et al [25] found that neuroticism is innate rather than caused by acquired environmental influences. Young children with neurotic traits (e.g., emotional impulsivity or lack of control) show high levels of negative emotions in their twenties. And a study by Gleason et al [26] assessed the life events of a neurotic sample of older adults at baseline through semi-structured interviews and questionnaires, and measured them again 6 months later. They found that those who exhibit a high degree of neuroticism are particularly prone to feel anxious or negative when facing stress, and that this phenomenon does not only occur in young neurotic people, but even persists into later life and negatively affects the lives of older people. However, these studies have focused on general emotional experiences in daily life and have not specifically explored the emotional responses of neurotic individuals in specific stressful situations (e.g., breakups). In particular, neurotic young adults lack mature ways of coping to handle major emotional setbacks and are especially prone to fall into negative emotions.

A number of related theories offer possible explanations for the strong link between neuroticism and negative emotions. For example, Gray’s [27] Reinforcement Sensitivity Theory (RST) suggests that increased sensitivity of neuroticism to punishment signals leads to an anxiety component, and that they tend to habitually retrieve negative information from memory and make negative judgments [28]. Specifically, neurotic individuals with the distinctive negative cognitive style seem inclined to recall negative memories [29]. They are more sensitive to negative experiences and are more prone to interpret even objectively ambiguous stimuli in a negative manner [30]. And from an etiological perspective, highly neurotic individuals have been found to have greater activation within the brain regions in charge of processing emotions, meaning that they need to make more effort in order to acquire cognitive management of negative feelings [31]. And high neurotic individuals are thought to maintain rigid and negative thinking habits, which makes them more prone to psychopathological symptoms [32]. Given the highly sensitive and susceptible nature of highly neurotic individuals when facing negative stimuli, their need to use a range of strategies for coping to regulate their emotional responses is particularly salient. Personality may directly influence the choice of coping strategies [33], and it is critical to investigate the coping strategies employed by neurotic people in facing distressing stressors, such as a romantic relationship breakup.

### Neuroticism and music listening coping strategy

Music is a form of communication in order to stimulate and convey emotions. It can help regulate emotions in many moments of life and is believed to promote or influence mental health [34,35]. In other words, the emotion regulation function of music is the attempt to reduce negative emotions, and maintain or increase positive emotions through music listening [36]. Many studies on neuroticism and music listening have shown that neuroticism is positively correlated with the emotional use of music (e.g., the use of music listening for emotion regulation), and this finding has been replicated in different cultural contexts, including Malaysia, Spain, the United Kingdom, and the United States [20,21,37,38]. Chamorro-premuzic and Furnham [37] attributed this phenomenon to the fact that those who are more neurotic tend to exhibit greater emotional instability and negativity. As a result of these emotional traits, they could be more susceptible to the emotional impact of music as well as more receptive to its influence [20].

Numerous researches in the domains of psychology, medicine, and music education have demonstrated that music serves as a significant modality for stress reduction [39,40] and triggers psychological, cognitive, and physiological responses that make it a balanced medium for handing stress [41]. In this technological era, listening to music has been recognized as a simple, affordable, and accessible method of coping and an effective strategy for managing drastic changes in one’s emotional state and distress [21,42]. However, research has found that music as an emotionally regulating self-management coping strategy exists in either adaptive (e.g., enhancing positive emotions) or maladaptive (e.g., venting negative emotions) forms [42,43]. As an example, Ahmadi [44] used semi-structured interviews to examine how individuals with cancer perceive music’s function as a way to cope. The findings suggested that music use was not universally beneficial and could, in some cases, be detrimental, occasionally exacerbating negative emotions. The potential detrimental effect of music use as a coping strategy has also been found elsewhere. For example, Saarikallio and colleagues [45] discovered that adolescents who use music maladaptively are more likely to experience depression and have worse mental health. However, when it comes to exploring the specific area of neurotic character traits and music listening coping, there is a relative paucity of relevant study, with only one 2-wave longitudinal study by Miranda et al [46], who followed 336 adolescents for six months and found that coping by listening to music as an escape or disengagement response may be a short-term risk precipitant for increased neuroticism in adolescence. It is therefore important to examine people’s specific traits to make sure music is employed in a beneficial way. Although studies have touched on the potential influence of music as a coping strategy in different populations, no research to date has specifically focused on how neurotic individuals use music listening as an adaptive or maladaptive coping strategy. Music serves a significant part in individuals’ daily lives as a universal and accessible coping resource. Understanding how neurotic individuals utilize music as a coping strategy and the potential impact of these strategies on their mental health can help aid in understanding the mechanisms of emotion regulation in neurotic individuals in specific contexts, and design more effective mental health education or interventions for this specific population.

### Music listening coping strategy as a potential mediator

According to Bolger and Zuckerman’s [47] model of personality and coping, when stressors are present, personality affects coping choices because personality influences how individuals react to stressful events. Many researches have revealed that individuals with maladaptive characteristics, including neuroticism, are more prone to utilize inappropriate ways to cope, such as avoiding strategies, and cause adverse outcomes [48,49]. Nevertheless, findings on the connection between coping styles and personality characteristics are inconsistent, with no significant correlations reported between the two [50]. Thus, there is a need to further explore how adaptive or maladaptive coping strategies act on emotion regulation processes in neurotic individuals.

As previously mentioned, neurotic individuals who are emotionally stressed and unstable prefer to use music to control their challenging moods due to their exceptionally sensitive nature to the emotional attributes of music [20]. Music as a rich cultural resource fascinates many young people, and music listening is often designated by young people as one of their key coping strategies [46]. Young people choose to cope by listening to music in a number of ways, including regulating their emotions to music, avoiding thinking about their stressors, and reflecting on solutions to stressful problems. Recent research suggests that neuroticism with traits of unstable emotional features may predict the usage of music listening as a method for managing moods [20]. Adaptive music listening, such as choosing pleasant or inspiring music to enhance positive emotions, can help alleviate unhappiness and anxiety and thus promote emotional recovery and mental health. In contrast, maladaptive music listening, such as seeking to vent negative emotions by repeatedly immersing oneself in sad or angry music, can lead to an exacerbation of the state of unhappiness, which can be detrimental to psychological well-being, even though the listener initially plans to utilize it as a healthy coping method [20,51].

Based to the psychological stress model developed by Lazarus and Folkman [19], the way people respond to stressful events simultaneously affects their emotions. Specifically, effective adaptive coping strategies can mitigate negative emotional reactions following a stressful event. For example, problem-focused coping strategies, which are efforts that attempt to directly solve a problem or change the situation, have been shown to help reduce long-term feelings of stress and enhance emotional well-being [52]. And some maladaptive coping strategies such as avoidance, substance abuse, or over-contemplation may provide temporary relief from distress. However, in the long run, they prevent individuals from effectively addressing the issues that are causing distress, thereby prolonging the state of negative affect [53]. The neurotic category is particularly vulnerable to negative emotions under the specific stressor of romantic relationship breakdown, making it particularly important to explore the effectiveness of specific coping strategies.

As stated earlier, the personality-cognition-emotion link can be inferred. That is, neuroticism as a personality trait may influence coping strategies, and coping styles may then influence individual emotions. In other words, it is reasonable to hypothesize that in the context of romantic relationship breakups, when love loss is a stressor, music listening coping may mediate the relationship between neuroticism and the negative emotions associated with a breakup. Unfortunately, this link has not been explored in the field of music. In addition, no study has yet investigated the relationship between neuroticism, adaptive-maladaptive music listening coping, and negative emotions associated with romantic relationship breakup. Given the difficulty of changing relatively solid and persistent personality features, for those with neurotic personalities experiencing the negative emotions of romantic relationship breakdown, it is necessary to explore whether and how neuroticism affects negative emotions through adaptive or maladaptive music listening coping, in order to be able to cope with them in a more positive way or to try to avoid exacerbating them. Thus, the present study concentrates on the specific mechanisms underlying two specific forms of coping strategies for music listening and the differences in their effects.

### The current study

To address these research gaps, the following three research questions were formed:

RQ1. Is neuroticism associated with adaptive music listening coping, maladaptive music listening coping and negative emotions associated with romantic breakups?

RQ2. Does adaptive music listening coping mediate the relationship between neuroticism and negative emotions of broken romantic relationships?

RQ3. Does maladaptive music listening coping mediate the relationship between neuroticism and negative emotions of broken romantic relationships?

Based on the above literature, the following three hypotheses were proposed:

H1. Neuroticism and negative emotions of broken romantic relationships are positively correlated with maladaptive music listening coping, while neuroticism and negative emotions of broken romantic relationships are negatively correlated with adaptive music listening coping.

H2. Adaptive music listening coping mediates the relationship between neuroticism and negative emotions of broken romantic relationships.

**Fig 1:**
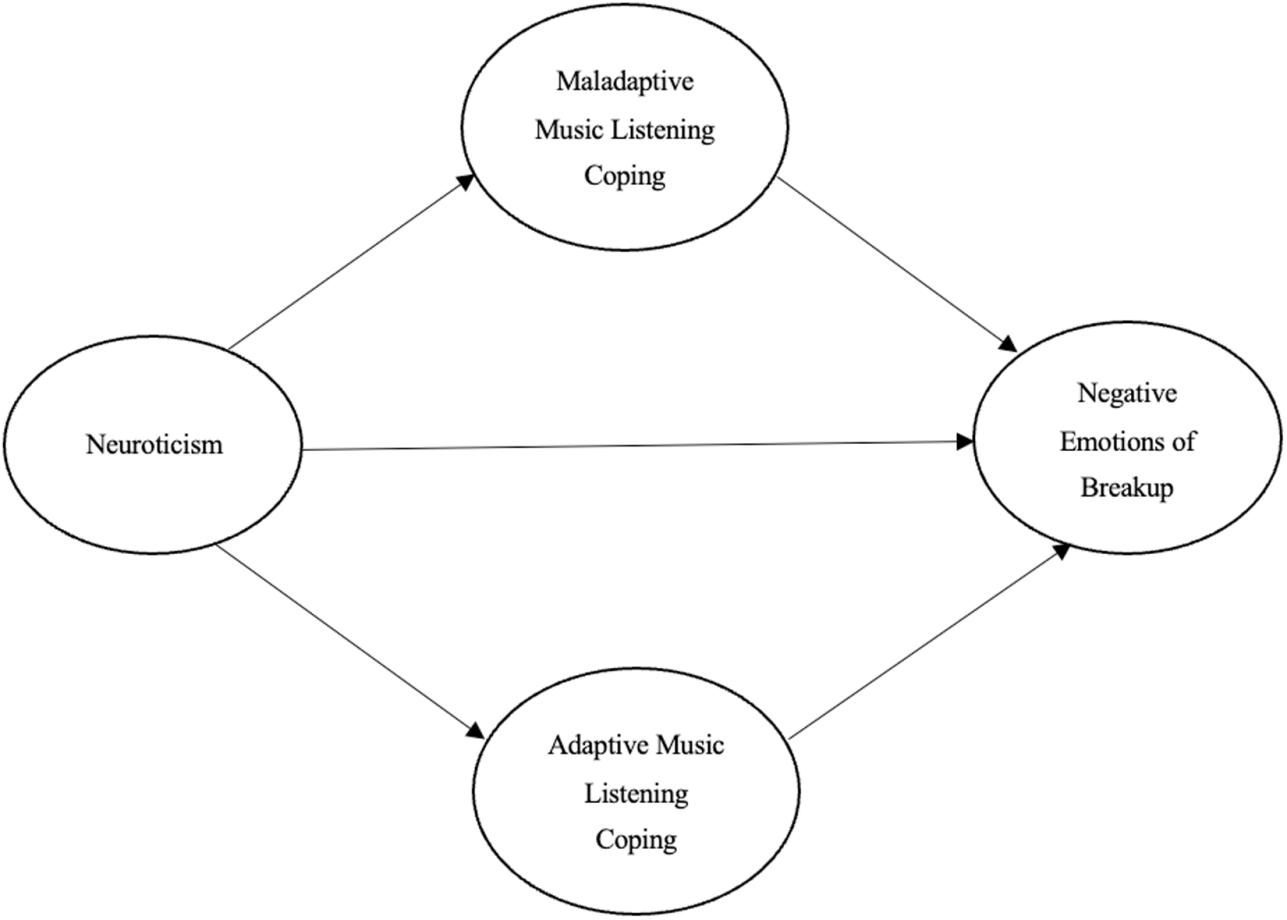
Diagram of the indirect effects model.

H3. Maladaptive music listening coping mediates the relationship between neuroticism and negative emotions of broken romantic relationships Fig 1.

## Materials and methods

### Participants and procedure

This study was conducted with a randomized sample at a normal university in southern China. Participants were recruited through email advertisements. In order to be eligible for participation, all participants in this research had to have undergone at least one romantic breakup lasting two months or longer within the past five years. The number of eligible participants was 467. After excluding invalid responses (incorrect answers to attention-checking questions, responses with certain traceable patterns, and response duration below M-2SD), 389 valid students (89 males; 300 females) were obtained with a mean age of years (M = 19.54; SD = 1.65). The corresponding author’s university Human Research Ethics Committee granted ethical permission for this study (Reference: CWNU.SRO/CWNUREC-186). The recruitment period began on 1st December 2023 and ended on 31st April 2024. Electronic questionnaires were created through a Chinese online survey platform (*Wenjuanxing*) and were distributed to participants via e-mail. Participants signed an online informed consent form and were informed of the purpose of the study and were fully aware of the confidentiality of the data collected and their right to withdraw from the study at any time without penalty. No monetary compensation was offered for the study Fig 1.

### Measurement

#### Neuroticism scale.

The Neuroticism Scale utilized the neuroticism subscale of the big five inventory, which was a personality trait scale developed by John and Srivastava [54]. The scale consists eight short phrase items and participants were requested to score phrases like “gets nervous easily” when describing themselves. The scale was scored from 1 to 5 (1 meant strongly disagree; 5 meant strongly agree), and scores for negatively phrased questions were reverse coded in this research. This questionnaire demonstrated acceptable reliability [55,56] with a Cronbach’s alpha value of.63.

#### Negative emotions of breakup.

The Negative Emotions of Breakup was used to measure the internal (sadness, anxiety) and external (anger) negative emotions that individuals experience during a breakup. It was based on research by Sprecher [57] and Twenge and Campbell [58] and adapted by Seidman and Schlott [59]. The scale consisted of three items and was rated on 7 points, where 1 represented not at all and 7 represented significantly. And participants were requested to recall their feelings at the time of the breakup to score sadness, anxiety, and anger. The scale demonstrated good internal consistency in the present research (Cronbach’s α = .76).

#### Adaptive- maladaptive music listening coping.

The Adaptive-Maladaptive Music Listening Coping used the Healthy-Unhealthy Music Scale (HUMS) designed by Saarikallio et al [45]. It consisted of two subscales with a total of thirteen items, where the HUMS Healthy Assessment was related to music use for experiencing social connection, relaxation, and positive emotions (sample item: “Music helps me to relax), and the HUMS Unhealthy Assessment was related to the use of music for contemplation, mood deterioration, and avoidance (sample item: “Music gives me an excuse not to face up to the real world”). The questionnaire was a 5-point Likert scale that asked participants to rate items by recalling their music use during a breakup (1 = never; 5 = always). Both subscales showed strong reliability in this research, with Cronbach’s α values of.90 and.89, respectively.

### Data analysis

A priori sample size analysis was first conducted in this research using G*Power software [60]. Three predictors were included, which were neuroticism, adaptive music listening coping and maladaptive music listening coping, with a medium effect size set at.15, a medium level of significance (α = .05), a statistical power of.95, and a minimum necessary sample size determined to be 119. Based on this power analysis, the present sample size of 389 was sufficient to test the hypothesized mediation model.

Descriptive statistics were first analyzed, including mean, standard deviation, kurtosis, and skewness, to confirm that the data met a normal distribution. Pearson’s correlation test was then used to analyze the correlations between the main variables. Finally, structural equation modeling (SEM) containing item packing was used to validate the proposed mediation model. In the SEM, maximum likelihood estimation (ML) was used to deal with missing data and chi-square values were used to assess the fit of the model. When the data fit the model well, then the chi-square value is not significant. Additionally, the model was assessed using the Tucker-Lewis index (TLI) and the comparative fit index (CFI), and the cutoff values were usually accepted as both TLI and CFI being larger than or equal to 0.9 [61]. Also, the root mean square error of approximation (RMSEA) and the standardized root mean square residual (SRMR) were also used to measure the fit of the model, with a cut-off value of ≤ 0.08 usually accepted for models [62]. Furthermore, a bias-corrected bootstrap test with a 95% confidence interval (CI) was used to test the statistical significance of the indirect effect [63]. All analyses were performed using SPSS 25 and Mplus 8.3 software.

## Results

### Descriptive and correlation analysis

Table 1 displayed the means, standard deviations, and Pearson’s correlation coefficients among the primary variables. According to the effect size (ES) criterion proposed by Cohen [64,65], the correlation coefficients |r| of.5,.3, and.1 represent large, medium, and small levels of correlation, respectively. Thus, neuroticism was moderately positively associated with maladaptive music listening coping (r = .330, p < 0.01), slightly negatively correlated with adaptive music listening coping (r = −.136, p < 0.01), and moderately positively correlated with negative emotions of breakup (r = .308, p < 0.01), and maladaptive music listening coping was moderately positively linked to negative emotions of breakup (r = .360, p < 0.01).

**Table 1:** Means, standard deviations and Pearson correlation between key variables (**N* *= 389).

Variable	M	SD	1	2	3	4
1. NEU	3.052	.526	1			
2. MMLC	2.062	.808	.330**	1		
3. AMLC	3.865	.777	−.136**	.016	1	
4. NEB	4.027	1.318	.308**	.360**	.004	1

NEU = Neuroticism; MMLC = Maladaptive Music Listening Coping; AMLC = Adaptive Music Listening Coping; NEB = Negative Emotions of Breakup; *p < 0.05, **p < 0.01.

### SEM analysis

An indirect effects model was employed to examine the effects of neuroticism, maladaptive music listening coping, and adaptive music listening coping on negative emotions of breakup. The model fit was acceptable (χ2 = 100.927, df = 41, SRMR = 0.043, RMSEA = 0.061 (95% CI: [0.046, 0.077]), TLI = 0.953, CFI = 0.965).

**Fig 2:**
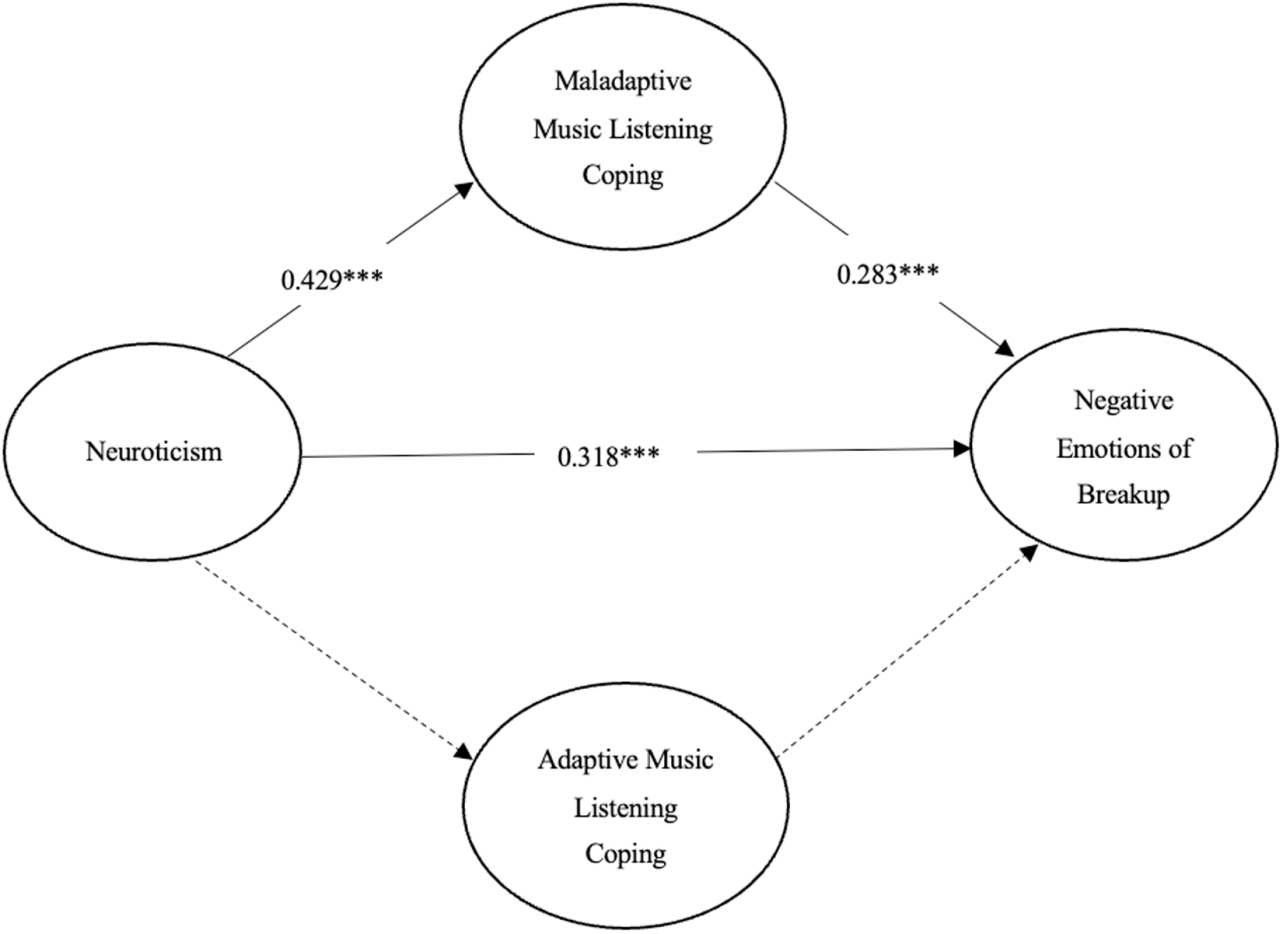
The indirect effects model with bias-corrected bootstrap test on mediating effects. *p < 0.05, ***p < 0.001.

As seen in Fig 2, neuroticism had a moderately significant direct effect on maladaptive music listening coping (β = 0.429, p < 0.001) and a moderately significant direct effect on negative emotions of breakup (β = 0.318, p < 0.001). And maladaptive music listening coping also had a moderate direct effect on negative emotions of breakup (β = 0.283, p < 0.001). However, adaptive music listening coping had no direct effect on negative emotions of breakup.

In addition to this, a bias-corrected bootstrap test with 95% CI was used to assess indirect effects in this study [63]. It could be seen in Table 2 that neuroticism had a slight indirect predictive effect on negative emotions of breakup through maladaptive music listening coping. That is, maladaptive music listening coping partially mediated the relationship between neuroticism and negative emotions of breakup (β = 0.121, p < 0.001, 95% CI: [0.079, 0.176]). And, there was no mediating role for adaptive music coping between neuroticism and negative emotions of breakup.

**Table 2:** Bias-corrected bootstrap test on mediating effects.

Paths	β	95% CI	
		Low	High
Neuroticism – Maladaptive Music Listening Coping	0.429***	0.323	0.512
Neuroticism – Negative Emotions of Breakup	0.318***	0.191	0.434
Maladaptive Music Listening Coping – Negative Emotions of Breakup	0.283***	0.173	0.383
Neuroticism – Maladaptive Music Listening Coping – Negative Emotions of Breakup	0.121***	0.079	0.176

***p < 0.001.

## Discussion

The aim of this research was to investigate whether neurotic personality was predictive of negative emotions of broken romantic relationships and whether this prediction was mediated by adaptive and maladaptive music listening coping. The results indicated that neuroticism and negative emotions of broken romantic relationships were positively correlated with maladaptive music listening coping, while neuroticism was negatively correlated with adaptive music listening coping. However, there was no relationship between adaptive music listening coping and negative emotions of broken romantic relationships. Thus, H1 was partially supported. In addition, only maladaptive music listening coping played a partial mediating role between neuroticism and negative emotions of broken romantic relationships. Therefore, H2 was partially supported and H3 was not supported.

The finding that neuroticism was positively associated with negative emotions of broken romantic relationships is consistent with previous research findings. Most studies have shown that neurotic individuals feel more distress when intimate relationships break down due to characteristics such as moodiness, anxiety, and depression [9,17,66]. China belongs to a typical collectivist culture that emphasizes introversion and stability. In this culture, individual emotional expression is often strongly influenced by social norms and group expectations [67]. Individuals in collectivist cultures are more inclined to suppress personal emotions than in individualistic cultures, especially in public or when confronted with family and society [68]. For highly sensitive neurotic individuals, this emotional suppression may exacerbate their internal negative emotional experiences. Therefore, in the Chinese cultural context, neurotic individuals may find it more difficult to alleviate their negative emotions through public expression of emotions after a breakup, leading to further deepening of emotional distress. The present study provides a new rationale for the generalizability of the relationship between neuroticism and negative emotions after a breakup by extending it to different cultural contexts. In addition to this, the existence of a correlation between neurotic personality and adaptive-maladaptive music listening coping is also in line with previous studies. During periods of high stress or negative emotion, individuals with neuroticism tend to use music listening to modulate their moods [20,21], and there are both adaptive and maladaptive forms of music listening as coping [43,51]. The present study builds on this foundation and further refines the relationship. In particular, in the specific context of romantic relationship breakdown, neurotic individuals may adopt both adaptive and maladaptive music listening strategies. This enriches our understanding of the musical preferences of neurotic individuals in difficult states and their psychological adjustment mechanisms.

Another noteworthy finding was a positive correlation between maladaptive music listening coping and negative emotions of broken romantic relationships, while no correlation existed between adaptive music listening coping and negative emotions of broken romantic relationships. In fact, the former finding is in line with the outcomes of some previous investigations. Negative emotions are exacerbated when people use maladaptive ways of music listening, such as venting and escaping through music [69]. This may be because disengagement and avoidance of stressors typically predict worse outcomes [33]. Listening to music to vent and escape unpleasant feelings may give people a false sense of mastery or assurance in the short term, but paradoxically, this can also subsequently cause negative emotions to become more intense and frequent [53]. Overreliance on these strategies can also narrow the scope of one’s behavior in response to naturally occurring bad moods, ultimately failing to override the negative beliefs that maintain the pattern [70]. However, the latter result also contradicts some research findings. Music, when used as an adaptive coping strategy, is often associated with reducing stress and improving mood [71,72], but this correlation was not found in the present study. This may be because, according to the Triple Vulnerability Model [73], which explains psychological disorders, the vulnerability specific to a breakup can cause a significant sense of uncontrollability by amplifying an individual’s negative emotions, ultimately leading to mood dysregulation [74]. Therefore, adaptive music listening coping may not be able to regulate such uncontrollable negative emotions caused by the loss of love. In addition to this, it may also be because maladaptive music coping strategies often involve rumination behaviors [75], which may reinforce an individual’s preoccupation with sadness, thereby exacerbating negative emotions and prolonging emotional recovery. In contrast, adaptive musical coping (e.g., listening to uplifting music) does not necessarily directly counteract negative emotions [76], but rather may serve as a neutral mechanism for emotion regulation, maintaining emotional balance without further worsening emotional states. And, adaptive coping strategies often imply a positive cognitive reappraisal of the breakup [77,78], which may not provide immediate relief from the negative emotions, but may contribute to long-term resilience. Maladaptive coping strategies, on the other hand, impede this cognitive reappraisal process, trapping individuals in a cycle of ongoing emotional distress. However, the existence of these exact situations warrants further research.

The results of SEM demonstrated the mediating role of maladaptive music listening coping, whereby neurotic individuals exacerbate the negative emotions of a breakup through maladaptive music listening coping. Moreover, it is worth noting that maladaptive music listening coping partially mediates the relationship between neuroticism and negative emotions of broken romantic relationships. This result is not unexpected. As stated earlier, not only does neuroticism directly predict negative emotions after a breakup [17], but given that neuroticism is often associated with maladaptive emotion regulation strategies [79,80], it is reasonable to conjecture that neuroticism would also be associated with maladaptive forms of musical emotion management [20], and this conjecture was validated in the present study. The present study further elucidates the mediating role of maladaptive music listening behavior in this process. This not only validates the strong link between neuroticism and negative emotions, but also reveals the underlying mechanism of action, which is the adoption of maladaptive mood control methods. Therefore, it is not difficult to imagine that young people with higher neuroticism traits tend to experience more dramatic emotional swings when a romantic relationship breaks down. This is because they are not yet fully mature in their emotion regulation abilities and are more sensitive to external stressors, and thus more inclined to rely on external resources (e.g., music) to regulate their emotions. When neurotic individuals choose maladaptive music listening strategies, negative emotions may be further exacerbated. The present study not only supports existing theoretical frameworks on neurotic personality and coping or emotion regulation, such as the Personality and Coping Model [47] and the Psychological Stress Model [19], but it also expands the scope of application of these theories. In particular, it reveals the complexity and diversity of emotion regulation strategies (especially music listening) of neurotic individuals when faced with a major life event (e.g., a breakup), which contributes to the understanding of how neurotic traits influence the choice of coping strategies and their effectiveness of individuals in stressful situations.

In addition to this, the importance of individualized interventions in mental health education for the college population is emphasized in this study. Given the relative stability and persistence of neurotic personality traits, a traditional uniform emotion management program may not meet the needs of all students. Therefore, when designing mental health education programs, educators should consider tailoring counseling programs to students with different personality traits. For example, for students with high neurotic tendencies, educators can focus on helping them identify and avoid maladaptive music listening strategies, such as repeatedly listening to songs that evoke sad emotions. At the same time, by introducing adaptive music strategies, such as music therapy to enhance positive emotions, these students can be helped to better manage mood swings and promote their mental health. And, mental health education programs for college students should incorporate education on the recognition of personality traits and coping strategies. By learning about the characteristics of different personality traits and their impact on emotional responses and behavioral patterns, students are able to better understand themselves and others. This deeper self-knowledge helps young people to understand their own strengths and weaknesses and to adopt more effective paths of personal development. Finally, the breakup of romantic relationships is a common experience among neurotic individuals. Through this study, students with higher levels of neuroticism should realize that they are more susceptible to negative emotions when experiencing stressors such as relationship breakup and learn to adopt proactive coping strategies. If they find it difficult to regulate their emotions effectively, they should seek the help of a professional counselor for more specialized guidance and support.

This study has some limitations. First, this study utilized a cross-sectional design and thus was unable to establish causal relationships between variables. To provide a firmer basis for causal inferences and to examine potential two-way interaction effects, a longitudinal design is necessary for future studies. Second, only students from a Chinese normal university participated in this study, and the proportion of female participants was higher than that of male participants, which may limit the generalizability of the research results. This may be because the actual enrollment structure of this normal university has a higher proportion of female students, which to some extent leads to a gender composition biased toward females among participants. Another possibility is that under the influence of hegemonic masculinity norms, men are often expected by society to exhibit traits of toughness and resilience, resulting in emotional expression inhibition [81]. As an emotional setback, breakups may be viewed by some men as personal failure or emotional vulnerability. Therefore, in order to maintain their self-image, males may tend to avoid emotional trauma and be reluctant to participate in related research surveys. Future studies should expand the sample size, especially by increasing the proportion of male participants, in order to improve the gender balance and generalizability of the results. Finally, this study relied heavily on self-reported data, an approach that may introduce a degree of subjective bias. In order to enhance the objectivity and reliability of future studies, it is recommended to integrate multiple data collection methods, such as behavioral observations and physiological measures, and other non-self-reported instruments, with the aim of providing a comprehensive and balanced assessment of the study participants. In this way, the complex relationship between individual differences and their coping strategies and emotional states can be more accurately captured.

## Conclusion

In summary, the present study explored the mediating mechanism between neuroticism and emotional response to breakup for the first time in the field of music. Specifically, this study found that people with neurotic personalities exacerbate their negative emotions after a breakup through the use of maladaptive music coping strategies, and that adaptive music coping strategies did not help them regulate their negative emotions. Therefore, because it may be difficult to change relatively stable and enduring personality traits, the use of maladaptive music coping strategies should be avoided or minimized for people with neurotic personalities when they are caught in a negative state after a breakup in order to reduce the severity of their negative emotions. This finding can provide new perspectives and references for mental health education and personal psychological adjustment. By guiding college students to understand their neuroticism trait levels and learn to recognize and use adaptive music listening strategies, it can help them understand how to manage their emotions more effectively when suffering from psychological distress due to life events such as loss of love.

## Supporting information

S1 File Original data. (XLSX)

S2 File Structural equation modeling results. (PDF)
