# Supplementary material for: Adaptive or maladaptive music-listening coping strategy: How does neuroticism use music after experiencing a romantic relationship breakup?
Source: PLoS One. 2025 Aug 29;20(8):e0331373. doi: 10.1371/journal.pone.0331373 (PMC12396676; doi:10.1371/journal.pone.0331373)
Supplement: S2 File — (PDF) [file pone.0331373.s002.pdf]

Mplus VERSION 8.3
MUTHEN & MUTHEN
09/19/2023   1:15 PM

INPUT INSTRUCTIONS

```
  DATA:
    FILE IS C:\Users\ThinkBook\Desktop\新建文件夹\389.dat;
  VARIABLE:
    NAMES ARE E1 E2 E3 MM1 MM2 MM3 MM4 Ne1 Ne2 Ne3 Ne4;
    Usevariables are E1 E2 E3 MM1 MM2 MM3 MM4 Ne1 Ne2 Ne3 Ne4;

          ANALYSIS:
            Type=General;
            Estimator=ML;
            Bootstrap=1000;

          MODEL:
            M BY MM1 MM2 MM3 MM4;
            E BY E1 E2 E3;
            N BY Ne1 Ne2 Ne3 Ne4;
            M on N;
            E on M;
            E on N;

          MODEL INDIRECT:
            E IND N;
          OUTPUT:
            Standardized Cinterval(Bcbootstrap);
```

INPUT READING TERMINATED NORMALLY

SUMMARY OF ANALYSIS

Number of groups	1
Number of observations	389
Number of dependent variables	11
Number of independent variables	0
Number of continuous latent variables	3

Observed dependent variables

Continuous

E1	E2	E3	MM1	MM2	MM3
MM4	NE1	NE2	NE3	NE4	

Continuous latent variables

M	E	N

Estimator	ML
Information matrix	OBSERVED
Maximum number of iterations	1000
Convergence criterion	0.500D-04
Maximum number of steepest descent iterations	20
Number of bootstrap draws	
Requested	1000

```
    Completed                                                     1000

Input data file(s)
  C:\Users\ThinkBook\Desktop\新建文件夹\389.dat

Input data format  FREE



UNIVARIATE SAMPLE STATISTICS


     UNIVARIATE HIGHER-ORDER MOMENT DESCRIPTIVE STATISTICS

         Variable/         Mean/     Skewness/   Minimum/ % with                Percentiles
        Sample Size      Variance    Kurtosis    Maximum  Min/Max      20%/60%    40%/80%    Median

     E1                     3.553      -0.163       1.000   14.40%       2.000      4.000      4.000
             389.000        2.222      -0.397       7.000    2.57%       4.000      5.000
     E2                     4.746      -0.651       1.000    7.20%       4.000      4.000      5.000
             389.000        2.632      -0.001       7.000   14.40%       5.000      6.000
     E3                     3.784      -0.105       1.000   14.40%       2.000      4.000      4.000
             389.000        2.863      -0.720       7.000    5.66%       4.000      5.000
     MM1                    3.913       0.823       2.000   28.02%       2.000      3.000      4.000
             389.000        2.918       0.518      10.000    1.03%       4.000      5.000
     MM2                    4.393       0.621       2.000   22.62%       2.000      4.000      4.000
             389.000        3.940      -0.305      10.000    1.29%       5.000      6.000
     MM3                    3.560       1.163       2.000   34.96%       2.000      3.000      3.000
             389.000        2.529       1.623      10.000    0.77%       4.000      5.000
     MM4                    4.630       0.591       2.000   16.71%       3.000      4.000      4.000
             389.000        4.105      -0.295      10.000    2.31%       5.000      6.000
     NE1                    5.871      -0.067       2.000    1.03%       5.000      6.000      6.000
             389.000        2.055      -0.012      10.000    0.26%       6.000      7.000
     NE2                    6.478      -0.172       2.000    1.80%       5.000      6.000      6.000
             389.000        2.902      -0.077      10.000    3.86%       7.000      8.000
     NE3                    5.491      -0.091       2.000    1.54%       5.000      5.000      6.000
             389.000        1.309       1.783      10.000    0.51%       6.000      6.000
     NE4                    6.578      -0.247       2.000    1.54%       6.000      6.000      6.000
             389.000        2.208       0.663      10.000    2.57%       7.000      8.000


THE MODEL ESTIMATION TERMINATED NORMALLY



MODEL FIT INFORMATION

Number of Free Parameters                       36

Loglikelihood

          H0 Value                      -7268.856
          H1 Value                      -7218.393

Information Criteria

          Akaike (AIC)                  14609.713
          Bayesian (BIC)                14752.402
          Sample-Size Adjusted BIC      14638.177
            (n* = (n + 2) / 24)

Chi-Square Test of Model Fit
```

Value	100.927	
Degrees of Freedom	41	1～3严谨
P-Value	0.0000	

RMSEA (Root Mean Square Error Of Approximation)　　＜0.08

Estimate	0.061		
90 Percent C.I.	0.046	0.077	
Probability RMSEA <= .05	0.104		

CFI/TLI

CFI	0.965	越接近1,＞0.95表示拟合优秀
TLI	0.953	

Chi-Square Test of Model Fit for the Baseline Model

Value	1770.525
Degrees of Freedom	55
P-Value	0.0000

SRMR (Standardized Root Mean Square Residual)　　＜0.08,＜0.05表示拟合优秀

Value	0.043

MODEL RESULTS

	Estimate	S.E.	Est./S.E.	Two-Tailed P-Value
M BY				
MM1	1.000	0.000	999.000	999.000
MM2	1.240	0.057	21.809	0.000
MM3	0.866	0.061	14.185	0.000
MM4	1.279	0.069	18.416	0.000
E BY				
E1	1.000	0.000	999.000	999.000
E2	1.455	0.187	7.761	0.000
E3	1.549	0.153	10.103	0.000
N BY				
NE1	1.000	0.000	999.000	999.000
NE2	1.701	0.254	6.709	0.000
NE3	0.769	0.123	6.267	0.000
NE4	1.099	0.146	7.512	0.000
M ON				
N	0.779	0.133	5.850	0.000
E ON				
M	0.174	0.046	3.815	0.000
N	0.356	0.097	3.684	0.000
Intercepts				
E1	3.553	0.075	47.520	0.000
E2	4.746	0.081	58.730	0.000
E3	3.784	0.083	45.683	0.000
MM1	3.913	0.086	45.623	0.000
MM2	4.393	0.100	43.778	0.000
MM3	3.560	0.082	43.503	0.000

```
    MM4                4.630      0.101     45.689      0.000
    NE1                5.871      0.073     80.158      0.000
    NE2                6.478      0.089     73.150      0.000
    NE3                5.491      0.061     90.098      0.000
    NE4                6.578      0.078     84.406      0.000

 Variances
    N                  0.596      0.138      4.324      0.000

 Residual Variances
    E1                 1.472      0.161      9.162      0.000
    E2                 1.044      0.170      6.135      0.000
    E3                 1.065      0.161      6.627      0.000
    MM1                0.950      0.099      9.633      0.000
    MM2                0.917      0.123      7.481      0.000
    MM3                1.052      0.114      9.247      0.000
    MM4                0.885      0.121      7.330      0.000
    NE1                1.459      0.136     10.742      0.000
    NE2                1.178      0.204      5.760      0.000
    NE3                0.957      0.109      8.786      0.000
    NE4                1.489      0.147     10.151      0.000
    M                  1.606      0.220      7.315      0.000
    E                  0.557      0.113      4.916      0.000


STANDARDIZED MODEL RESULTS


STDYX Standardization

                                                    Two-Tailed
                    Estimate       S.E.  Est./S.E.    P-Value

 M        BY
    MM1                0.821      0.024     34.902      0.000
    MM2                0.876      0.018     47.973      0.000
    MM3                0.764      0.031     24.644      0.000
    MM4                0.886      0.018     49.817      0.000

 E        BY
    E1                 0.581      0.054     10.736      0.000
    E2                 0.777      0.046     16.991      0.000
    E3                 0.793      0.034     23.036      0.000

 N        BY
    NE1                0.538      0.055      9.848      0.000
    NE2                0.771      0.044     17.594      0.000
    NE3                0.519      0.063      8.205      0.000
    NE4                0.571      0.059      9.695      0.000

 M        ON
    N                  0.429      0.055      7.778      0.000

 E        ON
    M                  0.283      0.064      4.435      0.000
    N                  0.318      0.072      4.398      0.000

 Intercepts
    E1                 2.384      0.096     24.916      0.000
    E2                 2.925      0.134     21.821      0.000
    E3                 2.236      0.085     26.363      0.000
    MM1                2.291      0.079     28.993      0.000
    MM2                2.213      0.067     32.869      0.000
```

```
    MM3                2.239      0.085     26.236      0.000
    MM4                2.285      0.070     32.614      0.000
    NE1                4.095      0.164     24.953      0.000
    NE2                3.802      0.156     24.397      0.000
    NE3                4.799      0.251     19.093      0.000
    NE4                4.427      0.202     21.956      0.000

 Variances
    N                  1.000      0.000    999.000    999.000

 Residual Variances
    E1                 0.662      0.062     10.635      0.000
    E2                 0.397      0.071      5.612      0.000
    E3                 0.372      0.054      6.839      0.000
    MM1                0.326      0.039      8.449      0.000
    MM2                0.233      0.032      7.290      0.000
    MM3                0.416      0.047      8.811      0.000
    MM4                0.216      0.031      6.864      0.000
    NE1                0.710      0.058     12.305      0.000
    NE2                0.406      0.067      6.028      0.000
    NE3                0.731      0.064     11.406      0.000
    NE4                0.674      0.066     10.205      0.000
    M                  0.816      0.046     17.677      0.000
    E                  0.742      0.053     14.064      0.000


STDY Standardization

                                                    Two-Tailed
                    Estimate       S.E.  Est./S.E.    P-Value

 M        BY
    MM1                0.821      0.024     34.902      0.000
    MM2                0.876      0.018     47.973      0.000
    MM3                0.764      0.031     24.644      0.000
    MM4                0.886      0.018     49.817      0.000

 E        BY
    E1                 0.581      0.054     10.736      0.000
    E2                 0.777      0.046     16.991      0.000
    E3                 0.793      0.034     23.036      0.000

 N        BY
    NE1                0.538      0.055      9.848      0.000
    NE2                0.771      0.044     17.594      0.000
    NE3                0.519      0.063      8.205      0.000
    NE4                0.571      0.059      9.695      0.000

 M        ON
    N                  0.429      0.055      7.778      0.000

 E        ON
    M                  0.283      0.064      4.435      0.000
    N                  0.318      0.072      4.398      0.000

 Intercepts
    E1                 2.384      0.096     24.916      0.000
    E2                 2.925      0.134     21.821      0.000
    E3                 2.236      0.085     26.363      0.000
    MM1                2.291      0.079     28.993      0.000
    MM2                2.213      0.067     32.869      0.000
    MM3                2.239      0.085     26.236      0.000
    MM4                2.285      0.070     32.614      0.000
```

NE1	4.095	0.164	24.953	0.000
NE2	3.802	0.156	24.397	0.000
NE3	4.799	0.251	19.093	0.000
NE4	4.427	0.202	21.956	0.000
**Variances**				
N	1.000	0.000	999.000	999.000
**Residual Variances**				
E1	0.662	0.062	10.635	0.000
E2	0.397	0.071	5.612	0.000
E3	0.372	0.054	6.839	0.000
MM1	0.326	0.039	8.449	0.000
MM2	0.233	0.032	7.290	0.000
MM3	0.416	0.047	8.811	0.000
MM4	0.216	0.031	6.864	0.000
NE1	0.710	0.058	12.305	0.000
NE2	0.406	0.067	6.028	0.000
NE3	0.731	0.064	11.406	0.000
NE4	0.674	0.066	10.205	0.000
M	0.816	0.046	17.677	0.000
E	0.742	0.053	14.064	0.000

STD Standardization

	Estimate	S.E.	Est./S.E.	Two-Tailed P-Value
**M BY**				
MM1	1.403	0.084	16.734	0.000
MM2	1.739	0.076	22.871	0.000
MM3	1.215	0.092	13.183	0.000
MM4	1.794	0.080	22.298	0.000
**E BY**				
E1	0.866	0.088	9.885	0.000
E2	1.260	0.101	12.528	0.000
E3	1.341	0.073	18.487	0.000
**N BY**				
NE1	0.772	0.090	8.546	0.000
NE2	1.313	0.091	14.383	0.000
NE3	0.594	0.085	7.000	0.000
NE4	0.848	0.107	7.962	0.000
**M ON**				
N	0.429	0.055	7.778	0.000
**E ON**				
M	0.283	0.064	4.435	0.000
N	0.318	0.072	4.398	0.000
**Intercepts**				
E1	3.553	0.075	47.520	0.000
E2	4.746	0.081	58.730	0.000
E3	3.784	0.083	45.683	0.000
MM1	3.913	0.086	45.623	0.000
MM2	4.393	0.100	43.778	0.000
MM3	3.560	0.082	43.503	0.000
MM4	4.630	0.101	45.689	0.000
NE1	5.871	0.073	80.158	0.000
NE2	6.478	0.089	73.150	0.000

NE3	5.491	0.061	90.098	0.000
NE4	6.578	0.078	84.406	0.000

Variances

N	1.000	0.000	999.000	999.000

Residual Variances

E1	1.472	0.161	9.162	0.000
E2	1.044	0.170	6.135	0.000
E3	1.065	0.161	6.627	0.000
MM1	0.950	0.099	9.633	0.000
MM2	0.917	0.123	7.481	0.000
MM3	1.052	0.114	9.247	0.000
MM4	0.885	0.121	7.330	0.000
NE1	1.459	0.136	10.742	0.000
NE2	1.178	0.204	5.760	0.000
NE3	0.957	0.109	8.786	0.000
NE4	1.489	0.147	10.151	0.000
M	0.816	0.046	17.677	0.000
E	0.742	0.053	14.064	0.000

R-SQUARE

Observed Variable	Estimate	S.E.	Est./S.E.	Two-Tailed P-Value
E1	0.338	0.062	5.420	0.000
E2	0.603	0.071	8.535	0.000
E3	0.628	0.054	11.552	0.000
MM1	0.674	0.039	17.497	0.000
MM2	0.767	0.032	24.032	0.000
MM3	0.584	0.047	12.370	0.000
MM4	0.784	0.031	24.963	0.000
NE1	0.290	0.058	5.026	0.000
NE2	0.594	0.067	8.828	0.000
NE3	0.269	0.064	4.203	0.000
NE4	0.326	0.066	4.931	0.000

Latent Variable	Estimate	S.E.	Est./S.E.	Two-Tailed P-Value
M	0.184	0.046	3.976	0.000
E	0.258	0.053	4.885	0.000

TOTAL, TOTAL INDIRECT, SPECIFIC INDIRECT, AND DIRECT EFFECTS

	Estimate	S.E.	Est./S.E.	Two-Tailed P-Value
Effects from N to E				
Total	0.492	0.100	4.906	0.000
Total indirect	0.136	0.041	3.341	0.001
Specific indirect 1				
E				
M				
N	0.136	0.041	3.341	0.001
Direct				

```
    E
    N                   0.356      0.097      3.684      0.000


STANDARDIZED TOTAL, TOTAL INDIRECT, SPECIFIC INDIRECT, AND DIRECT EFFECTS


STDYX Standardization

                                                    Two-Tailed
                    Estimate       S.E.  Est./S.E.    P-Value

Effects from N to E

  Total                 0.439      0.062      7.058      0.000
  Total indirect        0.121      0.030      4.017      0.000

  Specific indirect 1
    E
    M
    N                   0.121      0.030      4.017      0.000

  Direct
    E
    N                   0.318      0.072      4.398      0.000


STDY Standardization

                                                    Two-Tailed
                    Estimate       S.E.  Est./S.E.    P-Value

Effects from N to E

  Total                 0.439      0.062      7.058      0.000
  Total indirect        0.121      0.030      4.017      0.000

  Specific indirect 1
    E
    M
    N                   0.121      0.030      4.017      0.000

  Direct
    E
    N                   0.318      0.072      4.398      0.000


STD Standardization

                                                    Two-Tailed
                    Estimate       S.E.  Est./S.E.    P-Value

Effects from N to E

  Total                 0.439      0.062      7.058      0.000
  Total indirect        0.121      0.030      4.017      0.000

  Specific indirect 1
    E
    M
```

```
    N                    0.121      0.030      4.017      0.000

  Direct
    E
    N                    0.318      0.072      4.398      0.000
```

CONFIDENCE INTERVALS OF MODEL RESULTS

	Lower .5%	Lower 2.5%	Lower 5%	Estimate	Upper 5%	Upper 2.5%	Upper .5%
M BY							
MM1	1.000	1.000	1.000	1.000	1.000	1.000	1.000
MM2	1.117	1.147	1.163	1.240	1.345	1.379	1.418
MM3	0.717	0.747	0.764	0.866	0.969	0.992	1.042
MM4	1.117	1.145	1.167	1.279	1.395	1.423	1.459
E BY							
E1	1.000	1.000	1.000	1.000	1.000	1.000	1.000
E2	1.102	1.168	1.214	1.455	1.848	1.926	2.053
E3	1.242	1.301	1.353	1.549	1.865	1.946	2.064
N BY							
NE1	1.000	1.000	1.000	1.000	1.000	1.000	1.000
NE2	1.222	1.336	1.402	1.701	2.254	2.393	2.593
NE3	0.490	0.561	0.587	0.769	0.980	1.039	1.147
NE4	0.796	0.857	0.889	1.099	1.381	1.430	1.510
M ON							
N	0.521	0.563	0.592	0.779	1.027	1.077	1.213
E ON							
M	0.062	0.084	0.100	0.174	0.249	0.267	0.300
N	0.145	0.183	0.208	0.356	0.521	0.564	0.642
Intercepts							
E1	3.355	3.411	3.432	3.553	3.676	3.702	3.740
E2	4.506	4.568	4.617	4.746	4.874	4.900	4.956
E3	3.576	3.622	3.656	3.784	3.923	3.949	4.005
MM1	3.702	3.748	3.771	3.913	4.046	4.077	4.134
MM2	4.111	4.195	4.229	4.393	4.558	4.591	4.663
MM3	3.362	3.411	3.434	3.560	3.707	3.740	3.771
MM4	4.375	4.442	4.470	4.630	4.805	4.835	4.910
NE1	5.692	5.728	5.751	5.871	5.992	6.015	6.067
NE2	6.229	6.288	6.326	6.478	6.612	6.640	6.694
NE3	5.337	5.370	5.388	5.491	5.589	5.609	5.648
NE4	6.388	6.434	6.458	6.578	6.712	6.733	6.774
Variances							
N	0.269	0.339	0.374	0.596	0.823	0.892	0.981
Residual Variances							
E1	1.059	1.165	1.218	1.472	1.757	1.799	1.871
E2	0.627	0.722	0.764	1.044	1.333	1.387	1.478
E3	0.691	0.764	0.813	1.065	1.355	1.408	1.470
MM1	0.694	0.761	0.803	0.950	1.132	1.164	1.221
MM2	0.612	0.685	0.718	0.917	1.119	1.144	1.246
MM3	0.803	0.866	0.893	1.052	1.273	1.313	1.370
MM4	0.588	0.638	0.685	0.885	1.090	1.123	1.185
NE1	1.117	1.208	1.262	1.459	1.706	1.740	1.842
NE2	0.655	0.813	0.882	1.178	1.534	1.595	1.727

NE3	0.731	0.776	0.803	0.957	1.170	1.212	1.328
NE4	1.126	1.224	1.269	1.489	1.730	1.805	1.903
M	1.153	1.226	1.271	1.606	1.995	2.098	2.261
E	0.293	0.358	0.385	0.557	0.756	0.789	0.894

CONFIDENCE INTERVALS OF STANDARDIZED MODEL RESULTS

STDYX Standardization

	Lower .5%	Lower 2.5%	Lower 5%	Estimate	Upper 5%	Upper 2.5%	Upper .5%
M BY							
MM1	0.749	0.770	0.775	0.821	0.855	0.862	0.873
MM2	0.827	0.840	0.845	0.876	0.903	0.910	0.919
MM3	0.666	0.697	0.706	0.764	0.810	0.818	0.833
MM4	0.838	0.847	0.853	0.886	0.914	0.919	0.926
E BY							
E1	0.426	0.459	0.482	0.581	0.660	0.675	0.706
E2	0.646	0.676	0.697	0.777	0.847	0.859	0.884
E3	0.694	0.712	0.727	0.793	0.843	0.852	0.866
N BY							
NE1	0.365	0.409	0.435	0.538	0.616	0.632	0.665
NE2	0.617	0.673	0.691	0.771	0.836	0.846	0.871
NE3	0.321	0.379	0.401	0.519	0.611	0.625	0.647
NE4	0.405	0.443	0.465	0.571	0.659	0.677	0.700
M ON							
N	0.256	0.307	0.323	0.429	0.512	0.523	0.553
E ON							
M	0.109	0.146	0.173	0.283	0.383	0.407	0.439
N	0.129	0.172	0.191	0.318	0.434	0.455	0.491
Intercepts							
E1	2.146	2.205	2.232	2.384	2.545	2.583	2.641
E2	2.605	2.678	2.713	2.925	3.161	3.200	3.253
E3	2.026	2.075	2.099	2.236	2.379	2.420	2.459
MM1	2.098	2.142	2.175	2.291	2.430	2.457	2.505
MM2	2.040	2.086	2.106	2.213	2.327	2.346	2.393
MM3	2.036	2.077	2.097	2.239	2.378	2.409	2.464
MM4	2.119	2.160	2.175	2.285	2.407	2.433	2.472
NE1	3.670	3.786	3.819	4.095	4.361	4.411	4.497
NE2	3.454	3.522	3.571	3.802	4.079	4.128	4.228
NE3	4.157	4.324	4.370	4.799	5.218	5.304	5.483
NE4	3.906	4.051	4.107	4.427	4.758	4.824	4.938
Variances							
N	1.000	1.000	1.000	1.000	1.000	1.000	1.000
Residual Variances							
E1	0.497	0.544	0.564	0.662	0.767	0.789	0.812
E2	0.215	0.260	0.281	0.397	0.513	0.542	0.575
E3	0.243	0.274	0.290	0.372	0.469	0.490	0.517
MM1	0.237	0.257	0.269	0.326	0.399	0.407	0.434
MM2	0.154	0.173	0.184	0.233	0.286	0.293	0.313
MM3	0.306	0.331	0.344	0.416	0.500	0.512	0.543
MM4	0.143	0.154	0.164	0.216	0.272	0.281	0.296
NE1	0.557	0.600	0.620	0.710	0.811	0.833	0.860
NE2	0.237	0.284	0.301	0.406	0.523	0.540	0.597

NE3	0.575	0.608	0.627	0.731	0.839	0.854	0.895
NE4	0.509	0.541	0.564	0.674	0.783	0.804	0.836
M	0.694	0.725	0.738	0.816	0.896	0.905	0.929
E	0.612	0.639	0.656	0.742	0.827	0.837	0.857

STDY Standardization

	Lower .5%	Lower 2.5%	Lower 5%	Estimate	Upper 5%	Upper 2.5%	Upper .5%
M BY							
MM1	0.749	0.770	0.775	0.821	0.855	0.862	0.873
MM2	0.827	0.840	0.845	0.876	0.903	0.910	0.919
MM3	0.666	0.697	0.706	0.764	0.810	0.818	0.833
MM4	0.838	0.847	0.853	0.886	0.914	0.919	0.926
E BY							
E1	0.426	0.459	0.482	0.581	0.660	0.675	0.706
E2	0.646	0.676	0.697	0.777	0.847	0.859	0.884
E3	0.694	0.712	0.727	0.793	0.843	0.852	0.866
N BY							
NE1	0.365	0.409	0.435	0.538	0.616	0.632	0.665
NE2	0.617	0.673	0.691	0.771	0.836	0.846	0.871
NE3	0.321	0.379	0.401	0.519	0.611	0.625	0.647
NE4	0.405	0.443	0.465	0.571	0.659	0.677	0.700
M ON							
N	0.256	0.307	0.323	0.429	0.512	0.523	0.553
E ON							
M	0.109	0.146	0.173	0.283	0.383	0.407	0.439
N	0.129	0.172	0.191	0.318	0.434	0.455	0.491
Intercepts							
E1	2.146	2.205	2.232	2.384	2.545	2.583	2.641
E2	2.605	2.678	2.713	2.925	3.161	3.200	3.253
E3	2.026	2.075	2.099	2.236	2.379	2.420	2.459
MM1	2.098	2.142	2.175	2.291	2.430	2.457	2.505
MM2	2.040	2.086	2.106	2.213	2.327	2.346	2.393
MM3	2.036	2.077	2.097	2.239	2.378	2.409	2.464
MM4	2.119	2.160	2.175	2.285	2.407	2.433	2.472
NE1	3.670	3.786	3.819	4.095	4.361	4.411	4.497
NE2	3.454	3.522	3.571	3.802	4.079	4.128	4.228
NE3	4.157	4.324	4.370	4.799	5.218	5.304	5.483
NE4	3.906	4.051	4.107	4.427	4.758	4.824	4.938
Variances							
N	1.000	1.000	1.000	1.000	1.000	1.000	1.000
Residual Variances							
E1	0.497	0.544	0.564	0.662	0.767	0.789	0.812
E2	0.215	0.260	0.281	0.397	0.513	0.542	0.575
E3	0.243	0.274	0.290	0.372	0.469	0.490	0.517
MM1	0.237	0.257	0.269	0.326	0.399	0.407	0.434
MM2	0.154	0.173	0.184	0.233	0.286	0.293	0.313
MM3	0.306	0.331	0.344	0.416	0.500	0.512	0.543
MM4	0.143	0.154	0.164	0.216	0.272	0.281	0.296
NE1	0.557	0.600	0.620	0.710	0.811	0.833	0.860
NE2	0.237	0.284	0.301	0.406	0.523	0.540	0.597
NE3	0.575	0.608	0.627	0.731	0.839	0.854	0.895
NE4	0.509	0.541	0.564	0.674	0.783	0.804	0.836
M	0.694	0.725	0.738	0.816	0.896	0.905	0.929

E	0.612	0.639	0.656	0.742	0.827	0.837	0.857

STD Standardization

	Lower .5%	Lower 2.5%	Lower 5%	Estimate	Upper 5%	Upper 2.5%	Upper .5%
M BY							
MM1	1.209	1.233	1.261	1.403	1.531	1.564	1.612
MM2	1.547	1.581	1.606	1.739	1.860	1.886	1.917
MM3	0.993	1.051	1.074	1.215	1.379	1.410	1.475
MM4	1.605	1.641	1.665	1.794	1.925	1.951	1.999
E BY							
E1	0.621	0.669	0.708	0.866	1.001	1.025	1.075
E2	1.015	1.061	1.104	1.260	1.439	1.460	1.548
E3	1.127	1.179	1.206	1.341	1.444	1.463	1.497
N BY							
NE1	0.519	0.582	0.612	0.772	0.907	0.944	0.991
NE2	1.030	1.113	1.151	1.313	1.450	1.472	1.527
NE3	0.378	0.433	0.450	0.594	0.726	0.757	0.806
NE4	0.589	0.636	0.679	0.848	1.035	1.064	1.106
M ON							
N	0.256	0.307	0.323	0.429	0.512	0.523	0.553
E ON							
M	0.109	0.146	0.173	0.283	0.383	0.407	0.439
N	0.129	0.172	0.191	0.318	0.434	0.455	0.491
Intercepts							
E1	3.355	3.411	3.432	3.553	3.676	3.702	3.740
E2	4.506	4.568	4.617	4.746	4.874	4.900	4.956
E3	3.576	3.622	3.656	3.784	3.923	3.949	4.005
MM1	3.702	3.748	3.771	3.913	4.046	4.077	4.134
MM2	4.111	4.195	4.229	4.393	4.558	4.591	4.663
MM3	3.362	3.411	3.434	3.560	3.707	3.740	3.771
MM4	4.375	4.442	4.470	4.630	4.805	4.835	4.910
NE1	5.692	5.728	5.751	5.871	5.992	6.015	6.067
NE2	6.229	6.288	6.326	6.478	6.612	6.640	6.694
NE3	5.337	5.370	5.388	5.491	5.589	5.609	5.648
NE4	6.388	6.434	6.458	6.578	6.712	6.733	6.774
Variances							
N	1.000	1.000	1.000	1.000	1.000	1.000	1.000
Residual Variances							
E1	1.059	1.165	1.218	1.472	1.757	1.799	1.871
E2	0.627	0.722	0.764	1.044	1.333	1.387	1.478
E3	0.691	0.764	0.813	1.065	1.355	1.408	1.470
MM1	0.694	0.761	0.803	0.950	1.132	1.164	1.221
MM2	0.612	0.685	0.718	0.917	1.119	1.144	1.246
MM3	0.803	0.866	0.893	1.052	1.273	1.313	1.370
MM4	0.588	0.638	0.685	0.885	1.090	1.123	1.185
NE1	1.117	1.208	1.262	1.459	1.706	1.740	1.842
NE2	0.655	0.813	0.882	1.178	1.534	1.595	1.727
NE3	0.731	0.776	0.803	0.957	1.170	1.212	1.328
NE4	1.126	1.224	1.269	1.489	1.730	1.805	1.903
M	0.694	0.725	0.738	0.816	0.896	0.905	0.929
E	0.612	0.639	0.656	0.742	0.827	0.837	0.857

CONFIDENCE INTERVALS OF TOTAL, TOTAL INDIRECT, SPECIFIC INDIRECT, AND DIRECT EFFECTS

	Lower .5%	Lower 2.5%	Lower 5%	Estimate	Upper 5%	Upper 2.5%	Upper .5%
Effects from N to E							
Total	0.264	0.317	0.341	0.492	0.657	0.705	0.795
Total indirect	0.050	0.071	0.083	0.136	0.215	0.241	0.300
Specific indirect 1							
E							
M							
N	0.050	0.071	0.083	0.136	0.215	0.241	0.300
Direct							
E							
N	0.145	0.183	0.208	0.356	0.521	0.564	0.642

CONFIDENCE INTERVALS OF STANDARDIZED TOTAL, TOTAL INDIRECT, SPECIFIC INDIRECT, AND DIRECT EFFECTS

STDYX Standardization

	Lower .5%	Lower 2.5%	Lower 5%	Estimate	Upper 5%	Upper 2.5%	Upper .5%
Effects from N to E							
Total	0.287	0.309	0.334	0.439	0.536	0.556	0.589
Total indirect	0.051	0.065	0.079	0.121	0.176	0.191	0.224
Specific indirect 1							
E							
M							
N	0.051	0.065	0.079	0.121	0.176	0.191	0.224
Direct							
E							
N	0.129	0.172	0.191	0.318	0.434	0.455	0.491

STDY Standardization

	Lower .5%	Lower 2.5%	Lower 5%	Estimate	Upper 5%	Upper 2.5%	Upper .5%
Effects from N to E							
Total	0.287	0.309	0.334	0.439	0.536	0.556	0.589
Total indirect	0.051	0.065	0.079	0.121	0.176	0.191	0.224
Specific indirect 1							
E							
M							
N	0.051	0.065	0.079	0.121	0.176	0.191	0.224
Direct							
E							
N	0.129	0.172	0.191	0.318	0.434	0.455	0.491

STD Standardization

	Lower .5%	Lower 2.5%	Lower 5%	Estimate	Upper 5%	Upper 2.5%	Upper .5%
Effects from N to E							
Total	0.287	0.309	0.334	0.439	0.536	0.556	0.589
Total indirect	0.051	0.065	0.079	0.121	0.176	0.191	0.224
Specific indirect 1							
E							
M							
N	0.051	0.065	0.079	0.121	0.176	0.191	0.224
Direct							
E							
N	0.129	0.172	0.191	0.318	0.434	0.455	0.491

DIAGRAM INFORMATION

Use View Diagram under the Diagram menu in the Mplus Editor to view the diagram.
If running Mplus from the Mplus Diagrammer, the diagram opens automatically.

Diagram output
c:\users\thinkbook\desktop\mptext6.dgm

Beginning Time: 13:15:08
Ending Time: 13:15:10
Elapsed Time: 00:00:02

MUTHEN & MUTHEN
3463 Stoner Ave.
Los Angeles, CA 90066

Tel: (310) 391-9971
Fax: (310) 391-8971
Web: www.StatModel.com
Support: Support@StatModel.com

Copyright (c) 1998-2019 Muthen & Muthen
